# Comparison of robot versus fluoroscopy-assisted pedicle screw instrumentation in adolescent idiopathic scoliosis surgery: A retrospective study

**DOI:** 10.3389/fsurg.2022.1085580

**Published:** 2023-01-23

**Authors:** Canglong Hou, Huan Yang, Yu Chen, Yilin Yang, Beichen Zhang, Kai Chen, Ming Li, Mingyuan Yang, Kai Chen

**Affiliations:** ^1^ Department of Orthopedics, Shanghai Changhai Hospital, Shanghai, China; ^2^ Department of Orthopedics, Hua shan Hospital Affiliated to Fudan University, Shanghai, China; ^3^ Department of Rehabilitation, The First Rehabilitation Hospital of Shanghai, Shanghai, China

**Keywords:** adolescent idiopathic scoliosis, fluoroscopy, pedicle screws, robot, scoliosis surgery

## Abstract

The aim of this study was to explore whether a robot-assisted (RA) technique has advantages over the conventional fluoroscopy-assisted (FA) technique in clinical and radiological outcomes and whether it could decrease the incidence of mis-implantations of pedicle screws in adolescent idiopathic scoliosis (AIS) correction surgery. A total of 101 patients with AIS were recruited (RA group: 45 patients underwent RA screw insertion; FA group: 56 patients underwent FA screw insertion). When comparing the radiological data between the two groups, the major and secondary curves were both corrected proficiently with no difference in Cobb angle comparison at the last follow-up, suggesting that both the RA technique and the FA technique could lead to efficient radiographic correction and similar clinical outcomes (all, *p > *0.05). In the RA group, operation time, blood loss, and transfusion volume were significantly greater than those in the FA group, while the accuracy of screw implantations in patients with AIS with a thoracic scoliotic curve in the RA group was higher than that in the FA group. In conclusion, both the RA and FA techniques could approach proficient radiographic correction and similar clinical outcomes in AIS surgery. Compared with the conventional fluoroscopy technique, the RA technique might improve the accuracy of screw implantations in patients with AIS with a thoracic scoliotic curve, while the increased operation time, blood loss, and transfusion volume might be the disadvantages due to the preliminary stage of the learning curve.

## Introduction

Adolescent idiopathic scoliosis (AIS) is the most common spinal deformity, with a diverse prevalence in the range of 0.5%–5.2% in the pediatric population (1–3). In the past, the pedicle screw has been the predominant instrument in AIS correction surgery. However, screw misplacement has been constantly reported, with an incidence in the range of 20%–30%, 1% of which even resulted in severe neurovascular complications (4–6). Although robust preventative techniques, such as an intraoperative x-ray (7), CT (8), and an electrophysiology monitor (9), have been employed in AIS surgery, screw insertion-related complications still give rise to great challenges on the safety and satisfaction of AIS correction, such as injury of vessels and nerves. Therefore, how to decrease the incidence of mis-implantation of pedicle screws and prevent severe neurovascular complications remain challenges for spinal surgeons.

In recent years, robot-assisted technology has been widely adopted in the medical field, bringing a dramatic elevation of outcomes in surgery, which seems to provide perfect choices to reduce screw insertion-related complications and to obtain an ideal correction effect with radiation-free exposure for the surgeon (10–12). Croissant et al. (13) used a robot-assisted system for image-guided percutaneous K-wire insertion in minimally invasive interventions of the spine, and their results suggested that the robot assistance device performed with high accuracy and safety during instrumentation, without any perforation of the pedicle wall. A study by Shillingford et al. (14) demonstrated a robotic-assisted S2AI screw placement as safe, accurate, and reliable for achieving solid spinopelvic fixation, and there was no difference in the total screw implant accuracy between the free-hand and robot-assisted techniques (94.9% vs. 97.8%, *p = *0.630). Much data have been reported on the effectiveness and advancement of the robot-assisted technique in thoracic and lumbar spinal surgery (12, 15–19), while studies related to the correction of scoliotic curves usually accompanied with a dysplasia vertebrae pedicle are scarce. Therefore, it is essential to explore the accuracy of pedicle implantation using the robot-assisted technique in AIS correction surgery to avoid the disadvantages of pedicle mis-implantations, such as injury of vessels and nerves.

The aim of the present study was to explore whether the robot-assisted technique has advantages over the conventional fluoroscopy-assisted technique in clinical and radiological outcomes, and whether it could decrease the incidence of mis-implantation of pedicle screws in AIS correction surgery. We hope that our results provide theories of effectiveness of the robot-assisted technique in inserting accurate and secure pedicle screws in AIS surgery.

## Material and methods

### Patient recruitment

The study was approved by the ethics committee of our university (local ethics committee of Changhai Hospital, SMMU, No. CHEC2017–163). In accordance with the 1964 Helsinki declaration, informed consent was obtained from all participants or their parents or legal guardians when aged under 18 years. The inclusion criteria were as follows: (1) patients with AIS aged 10–18 years; (2) patients with a main curve Cobb angle more than 40° that needed posterior correction surgery; and (3) patients with complete medical records of anteroposterior and lateral full spinal x-ray, and preoperative and postoperative CT scans. Other types of scoliosis, such as neuromuscular scoliosis and syndrome scoliosis, were excluded from our study.

Whether patients with AIS would receive the Renaissance robot system or traditional fluoroscopy was determined randomly before surgery by the surgeons. The participants were divided into two groups according to whether surgery was conducted with the Renaissance robot system. A total of 45 patients underwent robot-assisted (Renaissance®; Mazor Robotics Ltd., Caesarea, Israel) correction surgery (RA group), while the other 56 patients underwent a pedicle screw insertion utilizing the free-hand technique assisted by traditional fluoroscopy (FA group). In order to further investigate the effect of robot-assisted technology on surgery, patients were further divided into subgroups on the basis of structural curve distribution: thoracic scoliosis (RA group 25, FA group 27) and thoracolumbar/lumbar scoliosis (RA group 20, FA group 29). Considering the minimum sample size estimation, we finally performed 46 cases in the RA group and 56 cases in the FA group as controls.

All surgical procedures were performed by one experienced surgical team in a single medical center. The patient population had a follow-up history of at least one consecutive year.

**Figure 1:**
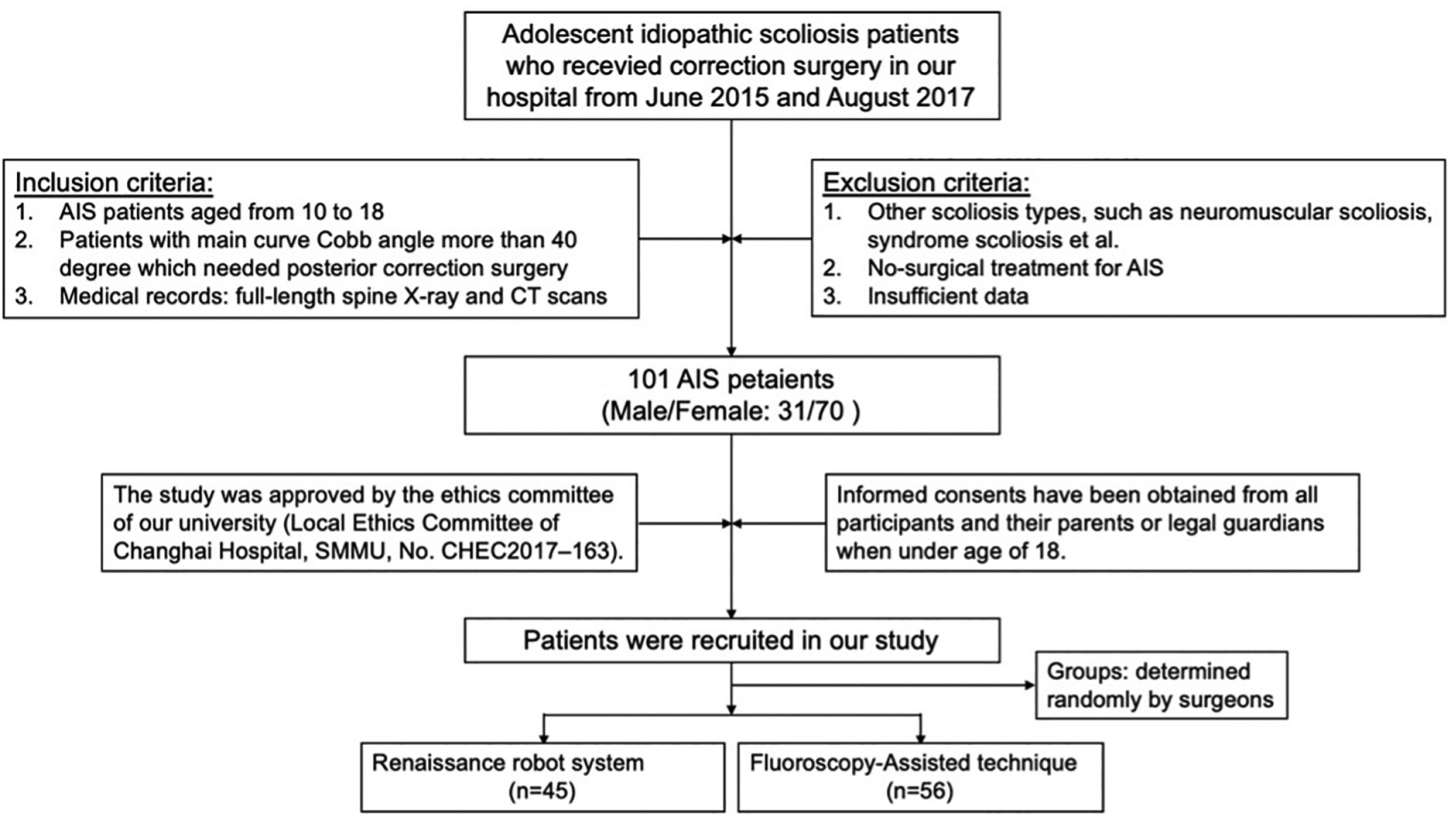
Flowchart showed the research methodology.

The research methodology is shown in Figure 1.

### Fluoroscopy-assisted technique

The patients were placed prone on a radiolucent surgery table and a posterior midline incision was made to expose the posterior elements at instrumented segments. According to the principles proposed by Kim et al. (20), the osseous anatomy was identified as a reference to choose screw entrance points. After penetration of the outer cortex using a high-speed burr, a pedicle probe was directed perpendicularly to the plane of the superior articular facet, and the medial and caudal inclination was adjusted to detect the safe screw trajectory (21). At the same time, intraoperative imaging was conducted by a C-arm to verify the exact vertebrae segment and position of the pedicle screws inserted in the coronal and sagittal planes. In addition, rod contouring and translation in situ bending were performed in subsequent correction procedures, as well as appropriate compression or distraction if necessary.

### Robot-assisted technique

**Figure 2:**
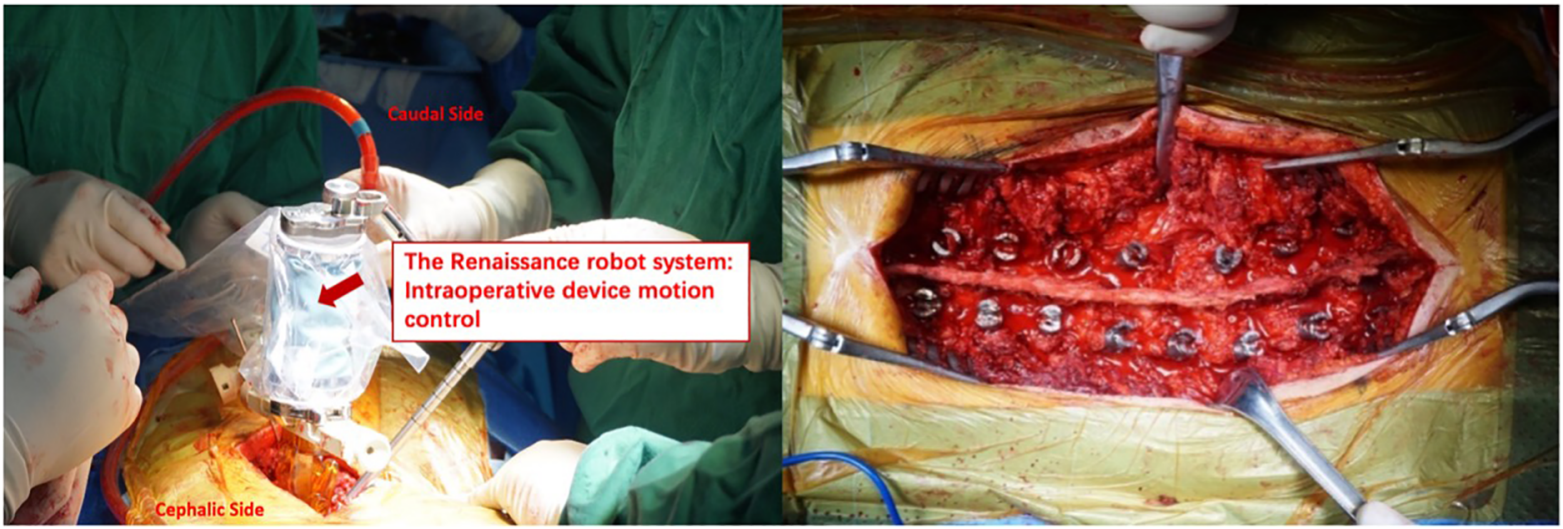
The controllable device of Renaissance robot system moves to planned screw trajectory position and direction on the basis of preoperative CT image, then pedicle screws were inserted according to the settled trajectory as drilled pathway.

The Renaissance robot system consists of a controllable robot device, a stabilized platform, and a surgical planning station for preoperative plan and intraoperative device motion control. Before the operation, the thin-cut (1 mm) CT scan data of the planned instrumentation segments were transferred into the software, and the inserted screw dimensions and position were confirmed according to the pedicle parameters. After mounting the stabilized platform to the spinous process, two intraoperative x-ray films were used to define each vertebrae segment location. The controllable robot device moved to the planned screw trajectory position and direction (shown in Figure 2), and the pedicle screws were inserted according to the settled trajectory as a drilled pathway. The later steps of the procedure were the same as those described in the fluoroscopy-assisted technique section above.

### Data collection

Baseline information was recorded, including age, gender, Risser sign, and Lenke types. Radiographic and clinical outcomes data were recorded in two groups preoperatively, immediately postoperatively, and at the last follow-up, including the major curve and secondary curve of the Cobb angle (the angle between the major curve and secondary curve), thoracic kyphosis (TK; the Cobb angle between T5 and T12), lumbar lordosis (LL; the Cobb angle between L1 and L5), coronal balance (CB; the horizontal distance between the center of the S1 vertebra and a vertical line drawn from the center of C7 and C7-CSVL), and sagittal vertical axis (SVA; the horizontal offset from the posterosuperior corner of S1 to the vertebral body of C7). The perioperative parameters, including operation time, blood loss, and transfusion volume during the operation, and postoperative complications were compared between the two groups.

A thin-cut (1 mm) CT scan was conducted postoperatively for all patients to evaluate the accuracy of the screw placement. The misplacement grading system introduced by Abul-Kasim et al. (22) classified screw malposition into five categories: medial cortical perforation (MCP); lateral cortical perforation (LCP); anterior cortical perforation (ACP); endplate perforation (EPP); and foraminal perforation (FP). In each category, grade 1 was identified with screws with partial cortical perforation, while grade 2 was identified as ACP and EPP as total cortical perforation.

Complications such as screw loosening, adding-on, proximal junctional kyphosis (PJK), and revision surgery were also studied and compared between the two groups. In addition, a SRS-22 questionnaire was adopted to evaluate the health-related quality of life (HRQOL) in our study.

### Statistical analysis

Statistical analyses were performed using SPSS 22.0 statistics software (IBM Corp., Armonk, NY, USA). Descriptive statistics were listed as the mean and standard deviation (SD), and categorical data were listed as numbers. Paired sample *t* tests were conducted to analyze the differences between the preoperative and postoperative parameters within the groups. The independent two-sample *t* test was used to compare the differences of the variables between the two groups. The chi-square test was used to compare the differences of count data. All statistical assessments were two-sided, and *p < *0.05 was considered to be statistically significant.

## Results

### Baseline information

A total of 101 patients with AIS (31 boys, 70 girls; mean age 14.58 ± 2.00 years) were recruited into our study. Of the patients, 45 (13 boys, 32 girls) were classified into the RA group, while the other 56 (18 boys, 38 girls) received the FA technique. No significant differences were observed in age, gender, Risser sign, and Lenke types between the two groups, as shown in Table 1 (all *p > *0.05). In addition, SRS-22 scores were also similar between the two groups (3.96 ± 0.52 vs. 3.93 ± 0.31, *p = *0.127).

**Table 1:** The baseline information, preoperative radiological parameters, surgery-related parameters, complications and HRQOL in two groups.

Variables	Group RA (*n* = 45)	Group FA (*n* = 56)	*P* value
*Baseline information*			
Age (years)	14.69 ± 1.93	14.49 ± 2.01	0.587
Gender (female/male)	32/13	38/18	0.725
Risser (^o^)	1.34 ± 0.90	1.41 ± 0.93	0.710
Lenke types (1/2/3/4/5/6)	22/8/4/1/7/3	25/10/4/0/13/4	0.823
*Preoperative radiological parameters*			
Cobb angle (^o^)			
Major curve	48.79 ± 7.03	47.14 ± 6.27	0.113
Secondary curve	27.83 ± 7.17	26.95 ± 5.67	0.512
TK	19.37 ± 3.78	20.30 ± 3.48	0.111
LL	39.02 ± 2.44	39.65 ± 2.50	0.159
CB (mm)	11.42 ± 2.13	11.36 ± 0.31	0.881
SVA (mm)	20.37 ± 3.42	20.44 ± 3.21	0.900
*Surgery-related parameters*			
Fusion level	10.41 ± 2.53	9.60 ± 2.84	0.094
Instrumented screws	14.51 ± 2.29	14.26 ± 2.07	0.464
Operation time (min)	210.12 ± 11.78	179.07 ± 16.60	**<0** **.** **001**
Blood loss (ml)	1063.07 ± 200.04	804.56 ± 137.17	**<0** **.** **001**
Transfusion volumes (ml)	946.09 ± 113.72	729.61 ± 147.56	**<0** **.** **001**
*Complications*			0.232
Screw loosening	2 (4.44%)	4 (7.14%)	
Adding-on	0 (0)	3 (5.36%)	
Proximal junctional kyphosis	3 (6.66%)	4 (7.14%)	
Revision surgery	1 (2.22%)	0 (0)	
*HRQOL*			
SRS-22 scores	3.96 ± 0.52	3.93 ± 0.31	0.127

Group RA, robot-assisted screw insertion group; Group FA, fluoroscopy-assisted screw insertion group; TK, thoracic kyphosis; LL, lumbar lordosis; CB, coronal balance; SVA, sagittal vertical axis; HRQOL, health-related quality of life.

### Comparisons of preoperative radiological parameters

The major Cobb angle, secondary Cobb angle, TK, and LL in the RA and FA groups were 48.79° ± 7.03°, 27.83° ± 7.17°, 19.37° ± 3.78°, and 39.02° ± 2.44°, and 47.14° ± 6.27°, 26.95° ± 5.67°, 20.30° ± 3.48°, and 39.65° ± 2.50°, respectively, with no significant difference (all *p > *0.05). In addition, we did not find any significant differences in CB and SVA, as shown in Table 1 (all *p > *0.05).

### Surgery-related parameters

With respect to the surgery-related parameters, the fusion level (10.41 ± 2.53 vs. 9.60 ± 2.84, *p = *0.094) and instrumented pedicle screw (14.51 ± 2.29 vs. 14.26 ± 2.07, *p = *0.464) in the RA group were comparable to those of the FA group. However, the operation time (210.12 ± 1.78 min vs. 179.07 ± 16.60 min, *p < *0.001), blood loss (1063.07 ± 200.04 ml vs. 804.56 ± 137.17 ml, *p < *0.001), and transfusion volumes (946.09 ± 113.72 ml vs. 729.61 ± 147.56 ml, *p < *0.001) in the RA group were greater than those in the FA group, suggesting the robot-assisted technique requires a longer operation time and consequently gives rise to more blood loss and a higher transfusion volume. In addition, the postoperative complications were also recorded in the 2 years of follow-up, and our results showed that the incidence of the screw loosening, adding-on, proximal junctional kyphosis, and revision surgery was 4.44%, 0, 6.66%, and 2.22% in the RA group, while it was 7.14%, 5.36%, 7.14%, and 0 in the FA group; no neurological complications occurred in the two groups. All the data are shown in Table 1.

### Radiological assessment of pedicle screw insertion

The evaluation of the accuracy of the pedicle screw instrumentation is shown in Table 2. In the RA group, 24 of 647 screws were classified into MCP, 32 were LCP, 4 were ACP, 3 were EP, and 2 were FP; while in the FA group, 34 of 771 screws were classified into MCP, 51 were LCP, 7 were ACP, 7 were EP, and 4 were FP. The total malposition rate of the RA and FA groups was 10.05% and 13.36%, respectively, and there was no difference between the two groups. While stratified into two types of scoliosis defined by structural curve distribution, the RA group demonstrated a significantly lower malposition rate in patients with thoracic scoliosis when compared with the FA group (89.70% vs. 84.4%, *p = *0.033), although there was no difference in the patient population with thoracolumbar/lumbar scoliosis (90.68% vs. 89.58%, *p = *0.454).

**Table 2:** Results of the radiological assessment of pedicle screw insertion between two groups.

	Group RA (*n* = 45)	Group FA (*n* = 56)	*P* value
**Total**			
Pedicle perforation	56 (8.66%)	85 (11.02%)	
Medial cortical perforation (MCP)	24 (3.71%)	34 (4.41%)	
Grade 1	16 (2.47%)	2 (3.11%)	
Grade 2	8 (1.24%)	10 (1.29%)	
Later Cortical perforation (LCP)	32 (4.95%)	51 (6.61%)	
Grade 1	22 (3.40%)	33 (4.28%)	
Grade 2	10 (1.55%)	18 (2.33%)	
Anterior cortical perforation (ACP)	4 (0.62%)	7 (0.91%)	
Endplate perforation (EP)	3 (0.46%)	7 (0.91%)	
Foraminal perforation (FP)	2 (0.31%)	4 (0.52%)	
Malposition screws	65 (10.05%)	103 (13.36%)	
Image satisfactory screws	584 (90.26%)	670 (86.90%)	
Sum up (all screws included in study)	647 (100%)	771 (100%)	0.054
**Thoracic scoliosis**			
Pedicle perforation	33 (8.94%)	49 (12.66%)	
Medial cortical perforation (MCP)	15 (4.07%)	19 (4.91%)	
Grade 1	12 (3.25%)	13 (3.36%)	
Grade 2	3 (0.81%)	6 (1.55%)	
Later Cortical perforation (LCP)	18 (4.88%)	30 (7.75%)	
Grade 1	12 (3.25%)	19 (4.91%)	
Grade 2	6 (1.63%)	11 (2.84%)	
Anterior cortical perforation (ACP)	3 (0.81%)	5 (1.29%)	
Endplate perforation (EP)	2 (0.54%)	4 (1.03%)	
Foraminal perforation (FP)	1 (0.27%)	2 (0.52%)	
Malposition screws	38 (10.30%)	60 (15.50%)	
Image satisfactory screws	331 (89.70%)	327 (84.50%)	
Sum up (all screws included in study)	369 (100%)	387 (100%)	**0** **.** **033**
**Thoracolumbar/lumbar scoliosis**			
Pedicle perforation	23 (8.24%)	36 (9.38%)	
Medial cortical perforation (MCP)	9 (2.23%)	15 (3.91%)	
Grade 1	4 (1.43%)	11 (2.86%)	
Grade 2	5 (1.79%)	4 (1.04%)	
Later Cortical perforation (LCP)	14 (5.02%)	21 (5.47%)	
Grade 1	10 (3.58%)	14 (3.65%)	
Grade 2	4 (1.43%)	7 (1.82%)	
Anterior cortical perforation (ACP)	1 (0.36%)	2 (0.52%)	
Endplate perforation (EP)	1 (0.36%)	3 (0.78%)	
Foraminal perforation (FP)	1 (0.36%)	2 (0.52%)	
Malposition screws	26 (9.32%)	43 (11.20%)	
Image satisfactory screws	253 (90.68%)	344 (89.58%)	
Sum up (all screws included in study)	279 (100%)	384 (100%)	0.454

Group RA, robot-assisted screw insertion group; Group FA, fluoroscopy-assisted screw insertion group.

### Comparisons of radiological parameters preoperatively and at final follow-up

When comparing the radiological data between the two groups, the major and secondary curve were both corrected proficiently; however, there was no difference in the Cobb angle of the major curve and secondary curve at the last follow-up. Although sagittal alignment (including TK and SVA) and coronal balance were significantly improved at the last follow-up in the RA and FA groups, the results showed no significant differences between the two groups. In addition, LL changed slightly from a mean of 39.02° ± 2.44° to 38.74° ± 7.05° in the RA group, and from a mean of 39.65° ± 2.50° to 35.95° ± 8.52° in the FA group, with no statistically significant differences between the two groups at the last follow-up.

The HRQOL scores remained stable in both the RA and FA groups, and showed no significant difference. The details were described in Table 3.

**Table 3:** Preoperative and last follow-up parameters between group RA and group FA.

	Group RA (*n* = 45)			Group FA (*n* = 56)			*P* value*
	Preoperative	Last follow-up	*P* value	Preoperative	Last follow-up	*P* value	
**Radiological Parameters**							
Cobb Angle							
Major Curve	48.79 ± 7.03	13.74 ± 5.11	**<0** **.** **001**	47.14 ± 6.27	12.97 ± 4.56	**<0** **.** **001**	0.445
Secondary Curve	27.83 ± 7.17	14.16 ± 4.40	**<0** **.** **001**	26.95 ± 5.67	12.91 ± 6.64	**<0** **.** **001**	0.119
TK	19.37 ± 3.78	24.98 ± 7.35	**<0** **.** **001**	20.30 ± 3.48	25.21 ± 4.52	**<0** **.** **001**	0.510
LL	39.02 ± 2.44	38.74 ± 7.05	0.818	39.65 ± 2.50	35.95 ± 8.52	0.257	0.286
CB (mm)	11.42 ± 2.13	7.53 ± 4.75	**<0** **.** **001**	11.36 ± 0.31	8.26 ± 5.34	**<0** **.** **001**	0.854
SVA (mm)	20.37 ± 3.42	7.03 ± 1.04	**<0** **.** **001**	20.44 ± 3.21	8.01 ± 5.96	**<0** **.** **001**	0.080
**HRQOL**							
SRS-22 Scores	3.96 ± 0.52	4.02 ± 0.48	0.342	3.93 ± 0.31	3.98 ± 0.54	0.215	0.118

Group RA, robot-assisted screw insertion group; Group FA, fluoroscopy-assisted screw insertion group; TK, thoracic kyphosis; LL, lumbar lordosis; CB, coronal balance; SVA, sagittal vertical axis; HRQOL, health-related quality of life.

* *P* value, *P* value of last follow-up parameters between group RA and group FA.

## Discussion

Due to the powerful three-column correction force, the pedicle screw has been widely applied in AIS correction surgery in recent decades. However, a relatively higher incidence of the malposition of pedicle screws has been reported by many researchers, which might result in severe neurovascular complications. AIS, as a three-dimensional (3D) spinal deformity, with its relative narrow pedicle and abnormality of vertebrae rotation, might be attributed to the high incidence of the malposition of pedicle screws, which may lead to the incidence of neurovascular complications. When compared with thoracolumbar/lumbar scoliosis, researchers found that the malposition of pedicle screws occurred more frequently in patients with thoracic scoliotic curves. De Blas et al. (23) claimed that the threshold magnitude of the thoracic pedicle was much lower than that of the lumbar pedicle. Pedicle screw insertion of the curve has been regarded as a great challenge for spinal surgeons, which is accompanied by the potential risk of vascular and neurological damage, especially in the population with a severe thoracic deformity. Therefore, it is necessary to explore techniques to decrease the mis-implantation of pedicle screws and prevent the occurrence of vascular and neurological damage.

Several pedicle screw insertion-assisted techniques, such as the 3D protype model and navigation system, have been widely applied in spinal surgery to reduce the incidence of screw malposition. Although the 3D protype model could provide a more comprehensive structure of the complex structure, the surgeons could not have access to gain instant information of the pedicles to adjust the directions and depth of the inserted screws during the operation (24). In addition, it has been reported that the accuracy of screw implantations of the navigation system was only 85% (25). More importantly, the limited improvement of these techniques may not far outweigh the complicated calibration procedures. Therefore, more effective and practical screw insertion-assisted techniques should be explored and applied in surgery to minimize the screw mis-implantation in AIS correction surgery.

The robot-assisted surgery technique, emerging as the new manipulation in spinal surgery, has been one of the most robust methods of improving the accuracy of screw insertions, and decreasing the risk of potential neurological complications and intraoperative radiation exposure since its introduction into clinical practice (13, 26, 27). In the study by Khan et al. (28), patients were divided into two distinct groups to compare the robotic technology with 3D CT navigation in degenerative disc diseases. One group consisted of 50 patients who underwent pedicle screw insertion guided by robot, and 189 of all 190 inserted pedicle screws were classified as Ravi I grade, and 1 screw as grade II. The other group consisted of 49 patients who underwent surgery with the assistance of a 3D CT navigation system, and 157 of all 165 screws were classified as Ravi grade I, and 8 screws as grade II. The results suggested that there was no significant difference in the field of screw insertion accuracy (*p = *0.11) between the groups, whereas the robot techniques could decrease the dose of radiation, time of per-screw insertion, and length of hospital stay compared to 3D CT navigation. In addition, many studies have demonstrated the superiority of the robot-assisted technique to conventional manipulations in general spinal surgeries. As far as we know, no study has been conducted to compare the clinical and radiological outcomes between the robot-assisted technique and conventional methods in AIS correction surgery, which might be huge challenges for spinal surgeons. The aims of this study were to explore the effectiveness of the robot system in AIS surgery, compare the accuracy of screw insertions between the robot and conventional fluoroscopy methods, and explore the radiological and clinical parameters.

In our study, there was no difference in the field of baseline information, preoperative radiographic parameters, fusion level, and number of instrumented screws (all, *p < *0.001) between the RA and FA groups, suggesting that the study populations of the two groups came from the same AIS cohorts and underwent correction surgery using the same strategy. Therefore, the selection bias and other biases that might impact our results, such as operation factors, were controlled. However, the operation time, blood loss, and transfusion volumes in the RA group were significantly greater than those of the FA group, which was consistent with the results of the studies by Le et al. (29), Ghasem et al. (30), and Fan et al. (31). This finding might result from the relatively complicated procedures of the robots compared with the FA group, such as the installation of a working panel, match with preoperative CT scans, and so on. However, Hyun et al. (32) reported a 1.5-min decrease in the per-screw insertion time between their first 15 robot-assisted cases and the last 15 cases. It seemed that the prolonged operation time was reduced as the number of total robot-assisted surgery cases rose, which might result from the effects of the robot learning curve (30). In our opinion, the operation time, blood loss, and transfusion volumes might decrease as our number of cases and experience increase, due to the learning curves.

With respect to postoperative complications, there were no significant differences observed between the two groups (*p = *0.232). With regard to HRQOL, there was no significant difference in the SRS-22 scores at the last follow-up between the two groups, showing the comparable clinical results in the RA and FA groups in the short follow-up durations.

With regard to the accuracy of the implantation of the pedicle screws, the total screw malposition rate was 10.05% in the RA group and 13.36% in the FA group, nearly approaching a significant difference between the two groups (*p = *0.054), suggesting that the robot-assisted technique could hardly claim to be significantly superior to conventional manipulations in radiological screw insertion accuracy, and the results were similar to those in the studies by Hyun et al. (32) and Park et al. (33). The malposition rate in our study was lower than those of other studies on degenerative disc diseases, which could be due to the dysplasia pedicle and vertebrae rotation in patients with AIS (32, 34, 35). In addition, the recruited patients in those studies might also influence the malposition rate of pedicle screws since each patient might have a unique structure of pedicles. When we stratified these patients with AIS into the thoracic scoliosis and thoracolumbar/lumbar scoliosis groups, our results showed that the robot-assisted technique could significantly decrease the malposition rate in AIS with thoracic scoliosis (*p = *0.033), whereas no significant difference in thoracolumbar/lumbar scoliosis subgroup was observed (*p = *0.454). The implantation of pedicle screws in thoracic curves is more difficult than that in thoracolumbar/lumbar scoliosis due to the smaller pedicles of the thoracic vertebrae and closer locations of vessels and nerves compared with that of the thoracolumbar/lumbar vertebrae. Therefore, in the RA group, the implantations of the pedicles were conducted using preoperative CT scans, which could provide more accurate information about the pedicles. In addition, the higher malposition rate of thoracic scoliosis in the FA group was attributed to more dysplastic vertebral morphometry in the thoracic segment than lumbar scoliosis, which was supported by the results in the studies by de Blas et al. (23), Shaw et al. (26), and Abul-Kasim et al. (36).

The major curve, secondary curve, TK, CB, and sagittal balance in patients with AIS were significantly corrected after surgery in both groups (all, *p < *0.001), and there was no difference in these postoperative parameters between the two groups. These findings suggested that both the robot-assisted and fluoroscopy-assisted techniques could reach proficient radiographic correction and similar clinical outcomes in AIS surgery. However, the LL in the RA group (*p = *0.818) and FA group (*p = *0.257) only showed a slight change after correction, and suggested no significant difference at the last follow-up in the two groups (*p = *0.286).

Although meaningful findings were observed and reported in our study, there were some potential limitations that should be addressed. First, all 101 patients with AIS were recruited from a single spinal surgery center, which might not represent all conditions of AIS correction surgery assisted by robot or fluoroscopy systems when considering the varied correction strategies and experiences on robot manipulation. In addition, the lack of long-term follow-up data, as well as the relatively small sample size of patients in our study, restricted the comparison conducted for further radiological and clinical outcomes. Therefore, persistent follow-up research with a larger population with AIS should be performed.

## Typical case

**Figure 3:**
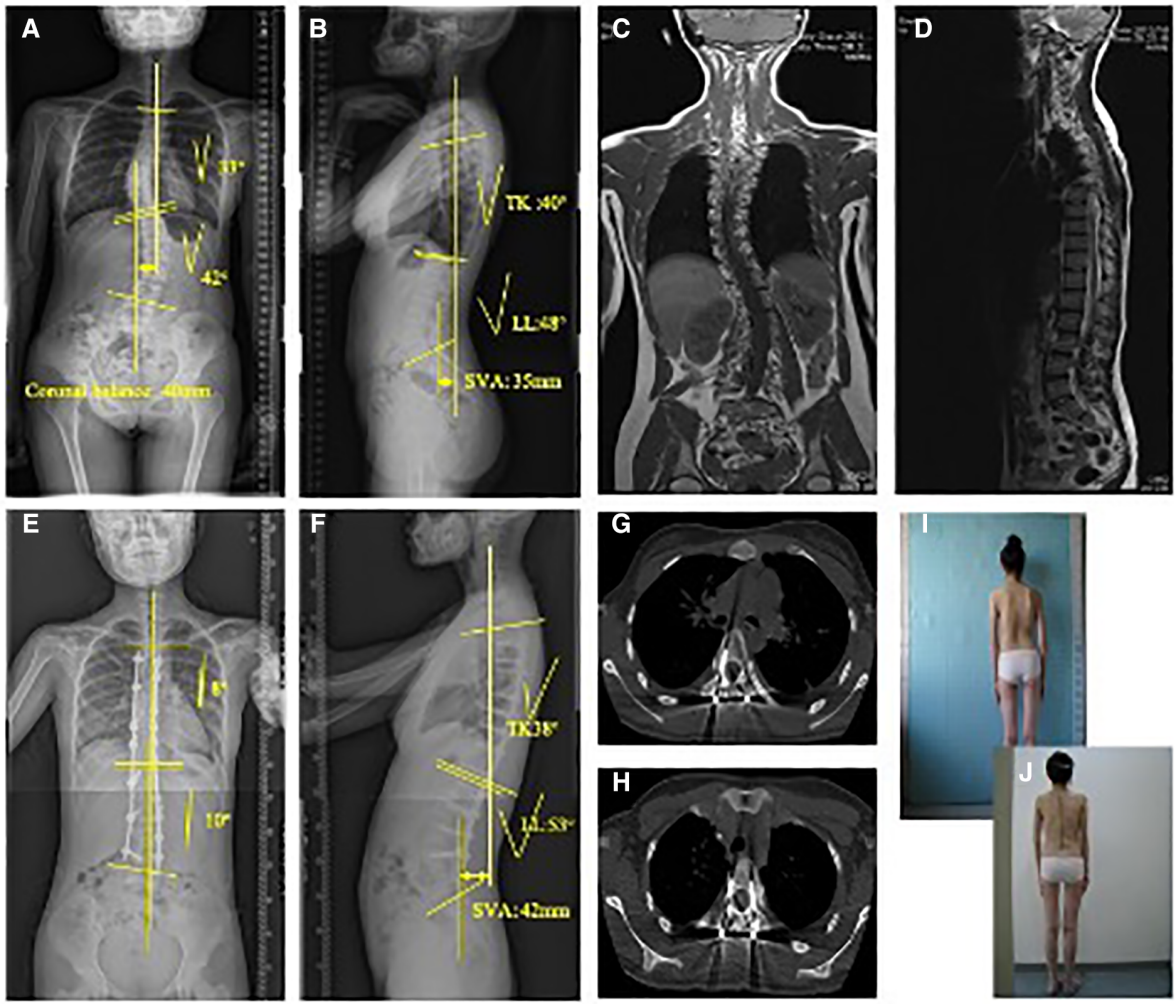
This picture showed the clinical pre-operative picture of this scoliosis patient. A 14-year old female Lenke 1 AIS patient underwent robot-assisted scoliosis correction surgery. Preoperative anteroposterior (**A**) and lateral (**B**) full spine X-ray showed a Cobb angle of 42°in major thoracic curve and 33°in secondary lumbar curve (the angle between major curve and secondary curve). Coronal (**C**) and sagittal (**D**) view of full spine MRI showed there was no abnormalities within spinal cord, which supported the diagnose of AIS when combined with other clinical characteristics. Postoperative anteroposterior (**E**) and lateral (**F**) full spine X-ray showed major thoracic curve was corrected to 10°, while secondary curve was corrected to 8°. Postoperative CT scan was used to evaluate the accuracy of screw insertion, and the typical image of satisfactory position (**G**) and lateral cortical perforation (**H**) were shown. In addition, clinical images of the patients both pre-operatively (**I**) and post-operatively (**J**) were also shown in Figure 3.

A typical case is presented in Figure 3.

## Conclusion

In conclusion, both the robot-assisted and fluoroscopy-assisted techniques could lead to proficient radiographic correction and similar clinical outcomes in AIS surgery. Compared with the conventional fluoroscopy technique, the robot-assisted technique might improve the accuracy of screw implantations in patients with AIS with a thoracic scoliotic curve, while increasing the operation time, blood loss, and transfusion volume during the operation due to the preliminary stage of the learning curve.

## Data Availability

The original contributions presented in the study are included in the article/Supplementary Material, further inquiries can be directed to the corresponding authors.
